# The Crusade against Physiological Experiment in England

**Published:** 1882-03

**Authors:** 


### EDITORIAL DEPARTMENT.

The Crusade Against Physiological Experiment in England.—For a number of years past, vivisection has been the subject of considerable acrimonious discussion in England. One of the results of this agitation has been the adoption of an Act of Parliament, requiring all persons proposing to experiment on living animals, to take out a license for this purpose. The license is issued at the discretion of the Home Secretary, who has it in his power to grant considerable latitude in the performance of the experiments, or to so hamper the action of the experimenter by restrictions as to make the license an absolute bar to the attainment of any useful result. Under this repressive law English physiologists have worked for the last four or five years, but as must be evident, under many disadvantages The opponents of vivisection are actively at work, are well organized and have officers of great influence with the laity. They continue agitating the subject with the view of prohibiting vivisection absolutely.

Miss Frances Power Cobbe, the Honorary Secretary, is a writer of considerable force, and uses her talent in the interest of the anti-vivisection society on every occasion. The constant attacks of the society upon physiologists, and the restrictions under which English experimenters worked, occasioned the expression of some very frank sentiments at the recent International Medical Congress by Professor Virchow and the venerable Mr. John Simon, to whom English sanitary science owes so much. According to Mr. Simon, the license Act has so much hampered the performance of physiological experiments in England that Professor Lister was compelled to go to France to perform certain experiments to elucidate an important point in pathology ; and Prof. Greenfield, who has already done so much for pathology, has been compelled to abandon his work altogether, because unwilling to submit to the restriction, annoyances and persecution which experimenters are compelled to submit to. We say persecution advisedly, for very recently Professor Ferrier was unceremoniously dragged before a magistrate, like any common criminal, because he had demonstrated before a medical audience the effect of the removal of certain portions of the brain in a monkey. On the investigation it appeared that the society had got hold of the wrong man, as Prof. Ferrier had not performed the experiments himself at all. Dr. Yeo, who had been the experimenter in this case was, however, protected by a proper license, and the society had no power to interfere with him. Of course Prof. Ferrier had to be acquitted, which seems to have aroused a very vindictive feeling in the anti-vivisectionist's camp, and they are now more active than ever.

In a recent number of the *Fortnightly Review* (January, 1882), Miss Cobbe, in replying to three papers, by Sir James Paget, Prof. Owen and Dr. Wilks, charges Sir James with "suppression of the truth and suggestion of the false," Prof. Owen with "direct misstatement," and Dr. Wilks with ignorance of the merits of the question he presumes to discuss.

As we said above, Miss Cobbe is a writer of considerable force, and the definite and determined manner in which she wipes out the history of physiological experiment and substitutes her own views, is calculated to make a much stronger impression upon the mind of the lay reader than the calm, dignified manner in which Sir James Paget, Mr. Simon, Professors Virchow, Owen and Humphry and Dr. Wilks have presented the claims of science and humanity in this matter. While we do not purpose to act the part of an alarmist, we cannot avoid calling attention to the necessity of a watchful care on the part of the profession in this country to promptly bring their influence to bear to counteract any covert attempts that may be made in the legislature of any of the United States, having for their object the enactment of similar repressive measures. The attempt to give significance to the recent indiscreet action of a number of medical students at a lecture by Mr. Henry Bergh in this city, and to create a popular feeling against physiological experimenters should be sufficient to warn us that the English anti-vivisectionists may soon have a following in this country sufficiently strong to interfere with the present gratifying position of scientific research amongst us. In this connection we refer to the recent admirable Cartwright lectures on "The Experimental Method in Medical Science," delivered by Prof. J. C. Dalton. Those of our readers who are not familiar with the history of physiological experiment and what it has done for the advancement of medical science should secure a copy of these excellent discourses in order that in any discussion of the question they may be in full possession of the facts to fortify their arguments.

Since the above article was put in type we have learned that an American anti-vivisection league had been formed in this city. This only furnishes an additional justification for the warning given above.

